# Somatic Embryogenesis in the *Medicago truncatula* Model: Cellular and Molecular Mechanisms

**DOI:** 10.3389/fpls.2019.00267

**Published:** 2019-03-19

**Authors:** Ray J. Rose

**Affiliations:** School of Environmental and Life Sciences, The University of Newcastle, Callaghan, NSW, Australia

**Keywords:** Auxin, cytokinin, ethylene, kinases, *M. truncatula*, somatic embryogenesis, totipotency, transcription factors

## Abstract

*Medicago truncatula* is now widely regarded as a legume model where there is an increasing range of genomic resources. Highly regenerable lines have been developed from the wild-type Jemalong cultivar, most likely due to epigenetic changes. These lines with high rates of somatic embryogenesis (SE) can be compared with wild-type where SE is rare. Much of the research has been with the high SE genotype Jemalong 2HA (2HA). SE can be induced from leaf tissue explants or isolated mesophyll protoplasts. In 2HA, the exogenous phytohormones 1-naphthaleneacetic acid (NAA) and 6-benzylaminopurine (BAP) are central to SE. However, there are interactions with ethylene, abscisic acid (ABA), and gibberellic acid (GA) which produce maximum SE. In the main, somatic embryos are derived from dedifferentiated cells, undergo organellar changes, and produce stem-like cells. There is evidence that the SE is induced as a result of a stress and hormone interaction and this is discussed. In *M. truncatula*, there are connections between stress and specific up-regulated genes and specific hormones and up-regulated genes during the SE induction phase. Some of the transcription factors have been knocked down using RNAi to show they are critical for SE induction (*MtWUSCHEL, MtSERF1).* SE research in *M. truncatula* has utilized high throughput transcriptomic and proteomic studies and the more detailed investigation of some individual genes. In this review, these studies are integrated to suggest a framework and timeline for some of the key events of SE induction in *M. truncatula.*

## Introduction

*Medicago truncatula* is a genetic and genomic model for legumes (Cook, 1999; Rose, 2008). *M. truncatula* has a small diploid genome, which is sequenced and annotated (Young et al., 2011; Tang et al., 2014), and a range of genetic and genomic resources are available (Benedito et al., 2008; Li et al., 2012; Rose, 2013; Garmier et al., 2017) as well as being represented in major data bases such as NCBI and Phytozome.

*M. truncatula* was first regenerated by somatic embryogenesis (SE) in 1989 (Nolan et al., 1989) and required a special seed line (Rose et al., 1999) called Jemalong 2HA (2HA). SE in wild-type Jemalong is rare. Subsequently, two other *M. truncatula* lines were developed that could also be regenerated by SE: R108 (Hoffmann et al., 1997) and M9-10a (Araújo et al., 2004). *M. truncatula* has predominantly been used to study nodulation but now is also used to study a wide range of plant biology, including the regulation of SE (Rose and Nolan, 2006). Unlike *Arabidopsis* (Mordhorst et al., 1998; Ikeda-Iwai et al., 2002; Harding et al., 2003; Kurczyńska et al., 2007; Kadokura et al., 2018) where primary somatic embryos come from immature embryos or seedling SAMs (shoot apical meristems), simple leaf explants can be used (Nolan et al., 1989; Nolan and Rose, 1998). In *M. truncatula*, an auxin plus cytokinin, rather than auxin alone, is required in the medium. Auxin alone in *M. truncatula* produces roots from procambium cells in the leaf explant veins (Rose et al., 2006). The specific auxin and cytokinin used vary between laboratories (Table 1). The absence of an exogenous cytokinin requirement for *Arabidopsis* represents a significant difference to *M. truncatula*. Interestingly, in the perennial *Medicago sativa,* SE can be produced by a pulse of 2,4-D in callus induced by an auxin and cytokinin (Dudits et al., 1991). In the 2HA line, abscisic acid (ABA) and gibberellic acid (GA) at appropriate concentrations and timing can stimulate SE over and above auxin + cytokinin (Table 1).

**Table 1:** Exogenous hormones and seed lines used for somatic embryogenesis.

Publications	Seed line, leaf explant	Auxin	Cytokinin	Other hormones
Nolan et al., 1989	2HA	NAA	BAP	
Chabaud et al., 1996	2HA	2,4-D	Zeatin or BAP	
Hoffmann et al., 1997	R108	2,4-D, NAA	BAP	
Nolan and Rose, 1998	2HA	NAA	BAP	ABA
Araújo et al., 2004	M9-10a	2,4-D	Zeatin	
Iantcheva et al., 2014	2HA cell culture	NAA	BAP	
Nolan et al., 2014	2HA	NAA	BAP	ABA+GA

**Figure 1:**
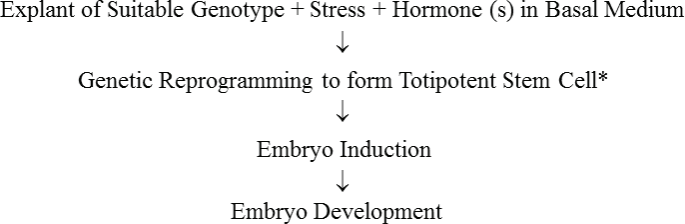
Sequence of steps in somatic embryogenesis. *It has been shown by tracking of a single labeled cell that embryos can develop from single somatic cells (Schmidt et al., 1997). A multicellular origin proposed by Williams and Maheswarin (1986) to occur in some cases has not been unequivocally demonstrated. Haccius (1978) concluded that somatic embryos can derive from a single cell or proembryonal cell complexes which are derived from a single segmenting cell.

In this review on SE in *M. truncatula*, the explant, the stress response, and the hormonal response, and how they are integrated in the generation of somatic embryos are considered (Figure 1).

## Explant of Suitable Genotype

### The Explant Genotype

For successful SE in *M. truncatula*, a special genotype is required. In the case of the three genotypes, all have been derived in a similar way, by selection after a cycle or cycles of tissue culture (Nolan et al., 1989; Hoffmann et al., 1997; Araújo et al., 2004). The ability of a cycle of tissue culture to consistently generate regenerable genotypes, which is heritable, is suggestive of epigenetic change as a result of the culture process. The 2HA genotype was developed from one of three rare Jemalong regenerates (Nolan et al., 1989). Each regenerate showed highly enhanced SE. Seed from the highest regenerator was selected for four generations to produce the 2HA line (Nolan et al., 1989; Rose et al., 1999). Earlier work on *M. sativa* had shown that regeneration capacity is genotype-specific, inherited, and could be enhanced by selection (Bingham et al., 1975; Reisch and Bingham, 1980). There is evidence that 2HA is an epigenetic variant of wild-type Jemalong. Amplified methylation polymorphism (AMP), an arbitrarily primed, methylation-sensitive PCR, showed many DNA methylation changes in 2HA, without detectable genome sequence change (Irwanto and Rose, 2008; Kurdyukov et al., 2014a). There are no obvious karyotypic differences between 2HA and wild-type Jemalong (Kurdyukov et al., 2014a). *MtEIL1*, an *EIN3-like* gene, is down-regulated and is methylated in the coding region. This methylation correlates with a small RNA that is antisense to the 3′ region. This gene is discussed further below. Another point of interest in the latter study is that two putative transposase genes, *BEDHAT1* and *BEDHAT2*, are up-regulated. These genes likely became hypomethylated (Kooke et al., 2015). It is not known if the other regenerable genotypes have similar DNA methylation changes. However, using 5-azacytidine with the highly regenerable M9-10a line to inhibit DNA methylation stopped somatic embryogenesis (Santos and Fevereiro, 2002).

### The Explant Cells—Leaf Explants

Leaf explants contain a number of different cell types in addition to the mesophyll cells. In the case of 2HA, the question of what cells are involved in SE has been examined (Wang et al., 2011). Most somatic embryos are derived from dedifferentiating mesophyll cells near the cut surface while some are derived from the vascular procambium. This was confirmed by changing the orientation of the explants. Vascularization can be greatly reduced by plating the leaf explant adaxial side down rather than abaxial side down and there is little difference in somatic embryo formation. While it is not possible to be unequivocal about why these two different cell types are involved, there are reasonable explanations based on previous literature with other species. The cells near the cut surface have ready access to wound stress molecules and stress can induce dedifferentiation (Grafi and Barak, 2015) and SE (Zavattieri et al., 2010; Rose et al., 2013; Fehér, 2015). The vascular procambium cells are stem-like cells and these types of cells are dedifferentiated and only require an SE-specific signal (Wang et al., 2011). A report in peach has also shown a procambial origin of somatic embryos (De Almeida et al., 2012).

The explants commonly used for investigation of SE in *Arabidopsis* are from immature zygotic embryos or the seedling SAM (Mordhorst et al., 1998; Gaj, 2001; Harding et al., 2003; Kurczyńska et al., 2007; Kadokura et al., 2018). Ikeda-Iwai et al. (2002) and Su et al. (2009) have used embryonic callus derived from primary embryos from immature zygotic embryos. Embryos develop from the edge of the callus. In general terms, the message from both *Arabidopsis* and *M. truncatula* and the wider literature is that SE can have an origin from uncommitted stem-like cells (Mordhorst et al., 1998; Rose, 2016) and cells that require dedifferentiation (Rose, 2016).

### The Explant Cells—Mesophyll Protoplasts

*M. truncatula* can form somatic embryos from isolated mesophyll protoplasts (Rose and Nolan, 1995). The isolated protoplasts form colonies that develop into callus. While embryos can initiate throughout the callus, it appears that embryos only develop fully when they approach the surface (Wang et al., 2011). This is consistent with the idea that it is peripheral cells of an explant, that may also be close to wounded cells as in leaf explants, that produce somatic embryos. Further, suitable auxin gradients may be easier to obtain near the surface of the callus, given auxin’s role in embryogenesis (Jenik and Barton, 2005).

With confocal microscopy and tracking organelles with fluorescent proteins, it is possible to visualize what happens to the organelles as the protoplasts form colonies. This has predominantly been carried out with *Nicotiana* and *Arabidopsis*, with some work on *M. truncatula*. There are three points of interest to emerge. Very early in culture, there is massive mitochondrial fusion in all three species (Sheahan et al., 2005), and in *Nicotiana* and *Arabidopsis* (not studied in *M. truncatula*), there are increases in peroxisomes (Tiew et al., 2015) and increases in P-bodies which are RNA processing bodies (Bhullar et al., 2017).

Massive mitochondrial fusion is indicative of preparation for a new generation (Rose and McCurdy, 2017) and is a response to the stress and hormones in the protoplast culture medium which ultimately leads to regeneration. The peroxisome proliferation is part of a stress response which is discussed further below. It can be argued that the increase in P-bodies reflects the degradation of transcripts characteristic of the differentiated cell, as it transits into cell division (Bhullar et al., 2017).

In analyzing the very first events in SE, it is important to distinguish between direct SE from stem-like cells and dedifferentiating cells developing embryonic callus as these early changes are different (Rose, 2016; Horstman et al., 2017a).

The *M. truncatula* system predominantly involves the dedifferentiation of cells and the formation of proembryogenic masses (PEMs) that have embryonic stem cells (Rose, 2016). Overall, the working hypothesis is that it is certain cells within the PEMs that have the right hormone environment to transit to SE formation. There is evidence for auxin gradients in the *Arabidopsis* callus, Su et al. (2009).

## SE and the Stress Response

**Figure 2:**
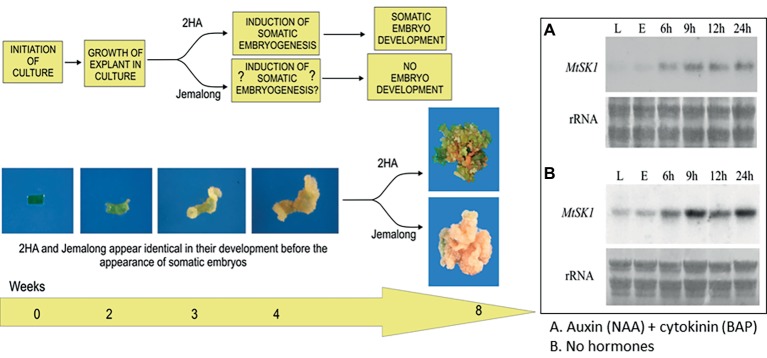
Interactions between stress and hormones in somatic embryogenesis. Development of explants from 2HA and wild-type Jemalong. Jemalong leaf explants develop into calli while 2HA explants develop into embryogenic calli capable of producing regenerated plants. Inset shows *MtSK1* expression (northern blots) in 2HA explants (E) from leaves (L) cultured for 24 h with and without hormones. Component figures reproduced from Nolan et al. (2006) with permission.

A proposed relationship between stress and hormones for *M. truncatula* is shown in Figure 2. In this context, the gene *Mt STRESS KINASE1* (*MtSK1*) has been investigated (Nolan et al., 2006). This gene is first expressed in the callus induction phase and is expressed in explants cultured in the presence or absence of the hormones auxin and cytokinin. MtSK1 is a stress-related kinase, responding to wounding and salt stress. Its close relationship to *SnRK2.4* of *Arabidopsis* and with no obvious responses to ABA (Nolan et al., 2006) like SnRK2 class 2 and 3 genes (Ng et al., 2014) suggests it is a class 1 SnRK2. How MtSK1 is connected to the SE response is not clear. However, there is an interesting relationship between class1 SnRK2 genes and the VARICOSE (VCS) mRNA decapping activator. VCS is the substrate for SnRK2 genes which subsequently causes decay of mRNA transcripts (Soma et al., 2017; Chantarachot and Bailey-Serres, 2018). In *Nicotiana* mesophyll protoplasts, VCS-containing P-bodies increase in the initial dedifferentiation phase of protoplast culture (first 48 h). It is plausible that MtSK1 and VCS are linked in dedifferentiation to remove transcripts associated with the dedifferentiation of the mesophyll explant cells.

**Figure 3:**
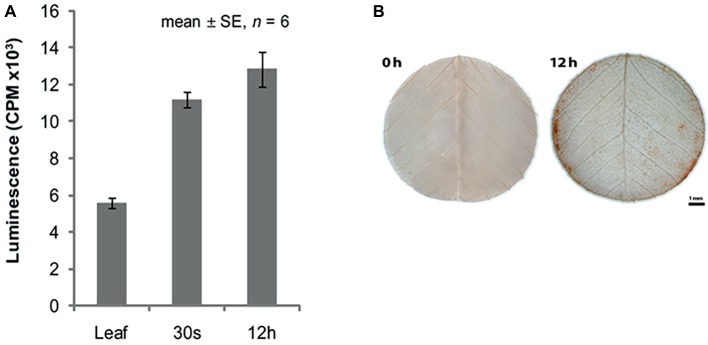
ROS in relation to somatic embryogenesis induction. **(A)** ROS production in leaf explants. **(B)** DAB (3,3'-diaminobenzidine) staining for H_2_O_2_. Bar = 1 mm. **(A,B)** reproduced with permission from Wang et al., 2011.

The increased expression of *MtSK1* occurs prior to 6 h after excision and plating, there are no data prior to 6 h. The very first change in the explant (Wang et al., 2011) is the production of reactive oxygen species (ROS), which occurs in seconds (Figure 3A). Soares et al. (2009) showed that after wounding *M. truncatula* leaves, there is an initial burst of O_2_^−^ for 0–30 min, which converts to H_2_O_2_ by superoxide dismutase. DAB (3,3′-diaminobenzidine) staining shows that the ROS is associated predominantly with the wound surface (Figure 3B), where most SE derives, with less staining associated with the vasculature (Wang et al., 2011). ROS can act as a signal (Mittler et al., 2011) but excessive ROS can be toxic (Foyer and Noctor, 2011). In quantitative proteomic studies of embryogenic 2HA versus wild-type Jemalong, enzymes involved in ROS detoxification were up-regulated: ascorbate peroxidase, thioredoxin h (TrnH), and peroxidoredoxin (Imin et al., 2005). This is consistent with ROS modulation in *M. truncatula* SE by up-regulation of redox genes and is supported by proteomic studies in other species (Heringer et al., 2018).

ROS homeostasis is essential for dedifferentiation and cell division induction, the beginning of callus formation (Fehér et al., 2008; Tiew et al., 2015). Zhang et al. (2018) have shown that thioredoxin regulates ROS homeostasis and *de novo* shoot regeneration in *Arabidopsis*. Excessive ROS is mitochondrially produced and inhibits shoot regeneration.

ROS inhibitors prevent SE induction in *M. truncatula* (Tiew, 2015). Whether there is a connection to MtSK1 is not known but it is an area that requires investigation. Mitochondria clearly produce a lot of ROS in the culture process that needs to be regulated (Tiew et al., 2015), but it is not the only source of ROS. ROS are produced by NADPH oxidases, encoded by respiratory burst oxidase homologs (*RBOHs*) in a plasma membrane complex. The *MtRBOHA* gene expression is up-regulated within the first week of culture, and is reduced by ABA+GA (Nolan et al., 2014), which increases SE, again suggesting the importance of modulation of ROS. In *M. sativa* protoplasts, ROS has been linked to auxin action and cell division induction in culture (Fehér et al., 2008). In *M. truncatula*, there is a link between ROS activity and ethylene production, which is discussed further in the sections below.

## The Role of Hormones in Relation to the Induction of Specific Genes

In understanding the SE process, it is ultimately necessary to understand the signaling processes involved and how this relates to both the hormones in the medium as well as endogenous hormones. While no doubt a number of parts of the process are similar across species, the detailed operation of the gene networks involved is likely to be species-specific. In the case of *M. truncatula*, the expression of some genes has been linked to specific hormones.

### *WUSCHEL* and Cytokinin

**Figure 4:**
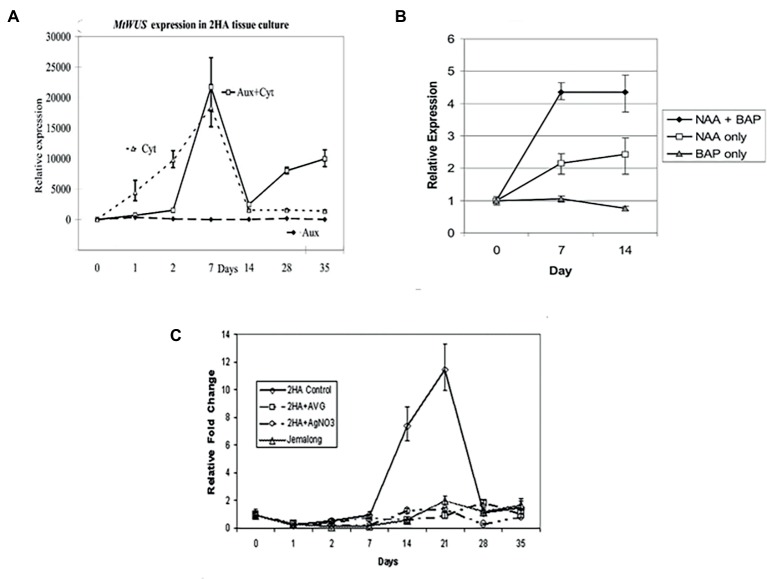
Effects of different hormones on somatic embryo induction. **(A)** *MtWUSCHEL* expression; **(B)** *MtSERK1* expression; **(C)** *MtSERF1* expression. AVG (10 μM aminoethoxyvinylglycine) and AgNO_3_ (10 μM) are ethylene biosynthesis and ethylene perception inhibitors, respectively. Aux = NAA = 10 μM 1-naphthalene acetic acid, Cyt = BAP = 4 μM 6-benzylaminopurine. **(A)** From Chen et al. (2009), authors’ copyright. **(B)** From Nolan et al. (2003), www.plantphysiol.org, Copyright American Society of Plant Biologists. **(C)** From Mantiri et al. (2008a,b), www.plantphysiol.org, Copyright American Society of Plant Biologists.

*MtWUSCHEL* is an ortholog of *AtWUSCHEL* hybridizing to the SAM stem cell niche (Chen et al., 2009) and in zygotic embryogenesis has the same time course expression as for *Arabidopsis* (Kurdyukov et al., 2014b). *WUSCHEL* (*WUS*) expression in *M. truncatula* is cytokinin dependent (Figure 4A), its expression increases a few days after excision, and RNAi studies have shown it is essential for SE (Chen et al., 2009). Similar results have also been shown in *Arabidopsis* (Su et al., 2009). However, in *Arabidopsis*, this early WUS expression in relation to SE is auxin dependent. Cytokinin in *Arabidopsis* cultures induces WUS expression and shoots (Gordon et al., 2007). In the case of *M. truncatula*, auxin alone produces numerous root primordia, from procambial cells, forming adventitious roots (Rose et al., 2006). Early *WUS* expression is characteristic of SE induction in *Arabidopsis, M. truncatula,* and *Brassica* (Chen et al., 2009; Su et al., 2009; Elhiti et al., 2010), consistent with the model of Fehér (2015). The well-established SAM expression occurs later as the bipolar embryo is formed (Mayer et al., 1998). This suggests that WUS in relation to SE can initiate an embryonic stem cell that progresses into embryogenesis. In *MtWUS*::GUS studies in *M. truncatula*, there are three expression stages: an initial phase throughout the early callus, then, when the explant is more fully callused, there are clusters of expression, and then embryo-associated expression. In *M. truncatula* zygotic embryogenesis, *WUS* expression also occurs in the ovule and early cell divisions of the embryo (Kurdyukov et al., 2014b), as it does in *Arabidopsis* (Groß-Hardt et al., 2002). It is feasible that the earliest expression found in 2HA, and also found in M9-10a but where there were no GUS studies (Orłowska and Kępczyńska, 2018), is more analogous to the ovule stage, the patches to PEMs, and subsequently the classic embryo *WUS* expression (Mayer et al., 1998). How then do these WUS expressing patches in the callus occur? The assumption is that this reflects parts of the callus where PEMs form as a result of suitable auxin and cytokinin concentrations. There are no data on this in *M. truncatula* but in the Su et al. (2009) study dealing with SE in *Arabidopsis* callus, *WUS* induction in patches is associated with *PIN-FORMED* (*PIN1*) expression and the setting up of appropriate auxin gradients. *In M. truncatula* 2HA, there is an initial peak expression of *WUS* at 7 d and then declines. *CLAVATA3* (*CLV3*) expression starts as the somatic embryo forms, establishing the WUS, CLV3 feedback loop characteristic of the SAM. A question that arises is given the role of WUS in the stem cells of the SAM, how can it be involved as an embryonic stem cell destined to form shoot and root meristems. Sarkar et al. (2007) have shown that WUS can act interchangeably with WOX5, the root stem cell maintenance gene. It was shown that a *WOX5-WUScDNA* transgene restored stem cells in the root meristem of a wox5 mutant.

### *SERK1* and Auxin

The *SOMATIC EMBRYO RECEPTOR-LIKE KINASE1* (*SERK1*) has been implicated in the induction of SE since its discovery by Schmidt et al. (1997) in carrot, where it is auxin-induced and expressed in cells destined to form somatic embryos. In *Arabidopsis*, overexpression of *SERK1* stimulates SE (Hecht et al., 2001) and is expressed to the early globular stage. *SERK1* is also expressed in ovules and early zygotic embryos. In *M. truncatula*, SERK1 is induced in both somatic embryo and root forming cultures (Nolan et al., 2003) in response to auxin. There is no response with cytokinin alone (Figure 4B). As noted earlier, auxin alone induces root formation. With auxin plus cytokinin, somatic embryos are induced and cytokinin acts synergistically with auxin to increase *MtSERK1* expression above auxin alone (Figure 4B). The *M. truncatula* results suggest that *SERK1* is not specific to SE or embryogenesis. In follow-up studies with MtSERK1 with promoter-GUS analysis, *MtSERK1* expression was found to be associated with developmental change (Nolan et al., 2009). There is expression of *MtSERK1* when callus is initiated and when somatic embryos are initiated. Expression is also associated with primary meristems of the shoot and root. The *M. truncatula* data do not indicate that SERK1 is not important in SE induction, rather that it is a gene connected to reprogramming of cells associated with developmental change. Studies with a range of species show *SERK1* expression is characteristic of early SE (Pandey and Chaudhary, 2014; Rocha et al., 2016). What is of interest in the case of *M. truncatula* is that cytokinin is key for *WUSCHEL* expression and auxin for *SERK1* expression.

### *SERF1* and Ethylene

The *MtSERF1* gene (*SOMATIC EMBRYO RELATED FACTOR1*) was discovered in the context of a cDNA microarray study in *M. truncatula* protoplasts at the transition stage, between callus and SE induction (Mantiri et al., 2008a). The microarray study showed up-regulation of ethylene biosynthesis and ethylene response genes. The *MtSERF1* gene is a member of the ERF sub-family of the AP2/ERF super family. It is up-regulated in 2HA but not wild-type Jemalong (Figure 4C). The expression of this gene peaks at 21d, the transition period between callus and somatic embryo production. The expression of the gene is ethylene dependent. The expression of this gene not only requires ethylene but is dependent on the presence of both auxin plus cytokinin (Rose and Song, 2018). This is a link between induced endogenous hormones and hormones supplied in the medium. What is the function of MtSERF1? There is some evidence that it is related to the action of WUS. This is based on the promoter region sequence of *MtSERF1* having WUS binding sites and the localization of *MtSERF1* expression to the apical region of the heart stage somatic embryo (Mantiri et al., 2008a).

As for auxin and cytokinin, the exact role for ethylene is species dependent, though it is clear that it is involved in SE (Kępczyńska and Zielińska, 2011; Fehér, 2015). The MADS box transcription factor AGAMOUS-LIKE15 (AGL15) stimulates SE when overexpressed in *Arabidopsis* and soybean (Zheng et al., 2013). AGL15 is able to stimulate ethylene production and *SERF1* expression (Zheng et al., 2013, 2016) suggesting that AGL15 could be associated with the stress response as well as modulating the auxin and GA response. There has been speculation on the targets of SERF1 in *M. truncatula* (Mantiri et al., 2008b) where the *HD-Zip III* genes *PHABULOSA, PHAVOLUTA,* and *REVOLUTA* were suggested, but there is no hard evidence on targets.

In *M. truncatula*, there is evidence that ethylene signaling is modified. Microarray studies by Imin et al. (2008) and more specific studies by Kurdyukov et al. (2014a) have shown that one of the two *EIN3-like* genes is down-regulated. This could possibly represent a necessary modulation of the ethylene response, preventing excess stress.

### *PICKLE*, GA, and ABA

The relationship of the *PICKLE* (*PKL*) gene to SE was discovered by Ogas et al. (1997). In the *pkl* mutant, cultured roots on basal medium could undergo SE without the application of plant hormones. SE in the *pkl* mutant is inhibited by GA. *PKL*, then, is a negative regulator of SE. This is conceptually important as the capacity for embryogenesis has to be switched off in somatic cells. If this gene is repressed, then the capacity for SE is enabled. The study by Zhang et al. (2008) shows that *PKL* and GA can act synergistically *via* separate pathways to repress expression of seed-associated genes. *PKL* is an ATP-dependent CHD3 chromatin remodeler which is part of complex that promotes the trimethylation of histone H3 lysine 27 (H3K27me3), a negative histone mark (Zhang et al., 2008). PKL represses expression of the embryo-specific transcriptional program, including the master regulators of embryogenesis LEAFY COTYLEDON1 (LEC1) and LEAFY COTYLEDON2 (LEC2), extensively studied in relation to SE (Zhang et al., 2012). It is not clear whether GA acts upstream or downstream of *LEC* genes in *PKL* repression of embryogenesis (Braybrook and Harada, 2008). In any event, models based on *Arabidopsis* data show that there is a nexus between *PKL, LEC1, LEC2, FUSCA3* (*FUS3*)*, AGL15*, GA, and ABA (Braybrook and Harada, 2008). Essentially, high ABA/GA ratios promote SE. However, in the case of *M. truncatula*, it is low ABA/GA ratios that promote SE and inhibit *PKL* (Nolan et al., 2014). Though ABA/GA ratios have not been investigated in *M. sativa*, the ABA and GA data alone are more similar to *M. truncatula* (Nolan and Rose, 1998; Ruduś et al., 2002; Ruduś et al., 2009; Nolan et al., 2014). This again shows how genes can be common to SE across species, but behave differently to plant hormones.

Some aspects of GA metabolism in relation to SE have been studied in *M. truncatula*. GA3 is synthesized in the SE induction period in the M9-10A embryogenic line (Igielski and Kępczyńska, 2017). GA in the medium inhibited SE in m9-10A, but at lower concentrations than in 2HA. In m9-10A, the GA biosynthesis inhibitor paclobutrazol also inhibited SE. This again suggests there are differences to *Arabidopsis* where this inhibitor promotes SE (Wang et al., 2004).

## Connecting Stress and Hormone Responses

In general terms, stress in the acquisition of SE has implicated 2,4-dichlorophenoxyacetic acid (2,4-D) at high concentrations, ABA, and ethylene (Karami and Saidi, 2010). Stress is involved in dedifferentiation (Grafi and Barak, 2015; Zhou et al., 2016) as well as in the activation of embryonic cell division (Pasternak et al., 2002; Rose et al., 2013). ROS are important in a plant’s response to stress (Chamnongpol et al., 1998; Podgórska et al., 2017). In the *M. truncatula* system (Tiew, 2015), as in *M. sativa,* ROS inhibition prevents SE (Fehér et al., 2008). In *M. sativa* protoplasts, ROS interact with auxin to initiate the cell cycle (Fehér et al., 2008). In cotton SE, there is an interplay between ROS and auxin to modulate SE (Zhou et al., 2016). Qu et al. (2017) have shown that H_2_O_2_ can regulate auxin distribution in lateral root development in *Arabidopsis* by regulation of *PIN2*. As shown in Figure 3B, ROS accumulate at sites where somatic embryos ultimately form.

Ethylene can be induced by ROS (Chamnongpol et al., 1998; Song et al., 2007) and is ROS dependent in *M. truncatula* (Tiew, 2015). In incubated excised mung bean hypocotyls, ROS promotes auxin-induced ethylene production (Song et al., 2007). Ethylene, together with auxin and cytokinin, is required for *MtSERF1* expression (Rose and Song, 2018). It is plausible that MtSERF1, with its requirement for ethylene, is a nexus between stress and auxin and cytokinin action (Mantiri et al., 2008a,b). In *Arabidopsis* and soybean studies, it has been shown that AGL15 stimulates expression of *SERF1* (Zheng et al., 2013), as well as *LEC2* (not tested in soybean), *FUS3*, and *ABSCISIC ACID INSENSITIVE3* (*ABI3*) genes which encode a B3 domain (Zheng et al., 2009; Zheng and Perry, 2014). *AGL15* expression has not been examined in *M. truncatula*, nor have the targets of SERF1. It would be expected that there would be some similarity to soybean where AGL15 not only stimulates *SERF1* but the *FUS3* and *ABI3* genes that are influenced by GA:ABA ratios (Braybrook and Harada, 2008; Zheng et al., 2009) and are required for embryogenesis. It is possible that ethylene influences GA action as well as auxin as discussed by Zheng et al. (2016). The conclusion from these latter studies in both soybean and *Arabidopsis* is that ethylene accumulation and response reduce the GA response facilitating SE. DELLA proteins may be significant in these interactions, where at least some ethylene response factors have been shown to interact with DELLA (Marín-de la Rosa et al., 2014). Ethylene biosynthesis and action have been shown to be important in the proliferation of embryogenic suspensions and embryo development in *M. sativa* L.cv. Rangelander (Kępczyńska et al., 2009, Kępczyńska and Zielińska, 2011) but not the initial induction (Kępczyńska et al., 2009).

## The Timeline for Metabolic and Gene Expression Changes in *M. Truncatula*

It is known from high throughput gene expression studies in legumes (Thibaud-Nissen et al., 2003; Imin et al., 2008; Mantiri et al., 2008a) that there are large numbers of gene expression changes associated with SE. However, there are a number of major genes that need to be put in perspective before the complexity of the integration of all the metabolic events associated with the developmental changes can be assembled. Genes or metabolites related to SE studied in *M. truncatula* and their approximate timeline are shown in Table 2. The different molecules and genes set out in Table 2 for *M. truncatula* can be discussed in the following framework.

**Table 2:** Sequence of changes of some key genes/metabolites in somatic embryogenesis.

*M. truncatula* Gene/metabolite	Type of molecule	Time to initiate (d)	Recorded peak (d)	Stage	References
ROS	e.g. H_2_0_2_	<1	0.5	Explant	Wang et al., 2011
*PKL* ↓(GA/ABA)	Chromatin remodeling ATPase	<7↓	14↓	Explant	Nolan et al., 2014
*PRC1* complex↓	Histone marks H2AK119ub↑	0–2↓	variable	Explant	Orłowska and Kępczyńska, 2018
*PRC2* complex (*CLF*, *MSI1*)*	Histone marks H3K27me3↑	2–7	14–21	Explant	Orłowska et al., 2017
*TRITHORAX* genes	Histone marks H3K4me3↑ H3K27ac↑ H3K26me2↑	<7	7	Explant	Orłowska and Kępczyńska, 2018
*MtSK1* (no hormones required)	SNRK2 kinase, class1	<6	35	Explant, embryogenic callus	Nolan et al., 2006
*TrxH*	Redox	<14	14	Explant	Imin et al., 2005
*WUS* (Cytokinin dependent)	TF (WOX family, stem cell maintenance)	3	7	Explant/callus/embryo	Chen et al., 2009
*STM*	TF (KNOX family, stem cell maintenance)	<7	7–14	Explant/callus/embryo	Orłowska and Kępczyńska, 2018
*SERK1* (Auxin dependent)	Receptor kinase	2–7	7	Explant/callus/embryo	Nolan et al., 2003, 2009
*AGL15*	TF (MADS box)	Not studied in *M. truncatula* but stimulates expression of *SERF1* in a legume - soybean. Zheng and Perry 2014			
*SERF1* (Ethylene dependent)	TF (ERF/AP2 family)	7–14	14–21	Explant/callus/embryo	Mantiri et al., 2008a,b
*BBM*	TF (ERF/AP2 family)	<7	7	Explant/callus/embryo	Imin et al., 2007; Igielski and Kępczyńska, 2017
*CLV3*	Peptide signal	14–28	35	Callus/embryo	Chen et al., 2009
*LEC1*	TF Master regulator encodes B3 domain	14–28	21	Callus/embryo	Nolan et al., 2014; Orłowska et al., 2017
*L1L*	TF master regulator encodes B3 domain	7–14	21	Callus/embryo	Orłowska et al., 2017
*WOX9*	TF (WOX family)	35	35	Early globular embryo	Kurdyukov et al., 2014b

*CLF is *CURLY LEAF*, *MSI1* is *MULTICOPY SUPPRESSOR OF IRA1*gene. Other gene abbreviations are indicated in the text. TF = transcription factor. Data from 2HA and M9-10A lines. ↓ = decreased gene expression, otherwise increased gene expression. ↑ = increased amount.

### Early Signals, Chromatin Remodeling, Dedifferentiation, and the First Cell Divisions

In cultured *M. truncatula*, ROS is the first signal as a result of the stress from the excision and plating of the explant. ROS is an important signal, but needs to be modulated as excess ROS can be toxic (Liu and He, 2016; Podgórska et al., 2017). Redox control then becomes essential and this is consistent with up-regulation of TrnH and ascorbate oxidase (Table 2, Imin et al., 2005). Increased number of peroxisomes also assists in redox homeostasis in this phase in *Arabidopsis* (Tiew et al., 2015). ROS can be generated by both NADPH oxidase and the electron transfer chain of mitochondria. There is some evidence that the initial ROS signal is due to NADPH oxidase (Soares et al., 2009) and *MtRBOHA* expression occurs before 7 d of culture (Nolan et al., 2014).

The transduction of the ROS signal together with the plant hormones in the medium initiate the chromatin changes leading to dedifferentiation and cell division initiation. The work of Zhao et al. (2001) shows isolated protoplasts undergo chromatin decondensation and there is increased DNA accessibility with propidium iodide. How this is linked to the stress/ROS/hormone interaction is unclear. Chromatin remodeling is influenced by PKL, POLYCOMB REPRESSIVE COMPLEX1 and 2 (PRC1 and PRC2), and the TRITHORAX GROUP proteins (TrxG) shown in Table 2. PKL contributes to H3K27me3 enrichment of loci, which is a repressive mark (Zhang et al., 2012), so down-regulation of PKL can facilitate derepression of genes required for SE. The PRC2 complex also increases H3K27me3 levels and is required for callus formation from leaf tissue where it represses the genes encoding leaf characteristics (He et al., 2012). The PRC1 complex ubiquinates histone H2A lysine 119 to compact chromatin (Schuettengruber et al., 2011; He et al., 2012) and needs to be down-regulated for SE. The up-regulation of *TRITHORAX* genes facilitates increased gene expression (Schuettengruber et al., 2011). Chromatin remodeling is clearly important in the transition to the dedifferentiated state where some genes need to be repressed and during SE where a number of genes need to be activated.

The specific role of MtSK1, characteristic of the excision and plating of the explant, in SE is not yet clear. However, given that it is a class 1 SnRK2 gene, implicated with RNA processing bodies, suggests a role in the degradation of transcripts from the original explant cells by interaction with P-bodies as the cells become meristematic (Soma et al., 2017; Chantarachot and Bailey-Serres, 2018). This degradation of existing transcripts characteristic of the explant leaf cells is critical to cell fate. The fusion of mitochondria appears to be important in ensuring that the integrity of the mitochondrial genome is maintained, to ensure cells have the capacity for regeneration (Rose and McCurdy, 2017).

### Callus and the Setting up of Stem Cells

Callus proliferation itself has not been studied in any detail in *M. truncatula* where the focus in the callus phase has been on the transition of callus cells to stem-like cells that will initiate embryos. In *Arabidopsis* WOUND INDUCED DEDIFFERENTIATION (WIND) transcription factors, members of the AP2/ERF transcription factor family have been shown to be important regulators of wound-induced callus (Iwase et al., 2011; Ikeuchi et al., 2017). The *WUSCHEL*-RELATED HOMEOBOX5 (WOX5) root meristem transcription factor is important in callus induction from pericycle cells in response to 2,4-D and kinetin in *Arabidopsis* (Sugimoto et al., 2010). In *M. truncatula*, WOX5 expression is high in explants cultured with auxin where callus and root primordia come from procambium cells (Chen et al., 2009). Less WOX5 expression occurs in the auxin + cytokinin medium (Chen et al., 2009; Orłowska and Kępczyńska, 2018), with massive callus coming from dedifferentiation of mesophyll cells rather than the procambium (Chen et al., 2009), and is where SE mainly occurs.

Early expression of *WUS* is characteristic of SE (Fehér 2015; Mahdavi-Darvari et al., 2015) and there is a good case that it is a critical gene required for the production of embryonic stem cells (Zuo et al., 2002; Chen et al., 2009; Su et al., 2009; Elhiti et al., 2010). In *M. truncatula* SE from leaf explants, the very early expression of WUS is more analogous to the ovule expression. Subsequently, the induction of *CLV3* is important in forming patches of WUS expressing cells (Mantiri et al., 2008a) in the callus, similar to patches of expression in *Arabidopsis* embryogenic callus (Su et al., 2009). These patches of WUS expression could correspond to the densely cytoplasmic cells of the PEMs. Su et al. (2009), in *Arabidopsis*, have shown that auxin gradients and *PIN* genes are required for WUS expression in SE, but the location of PIN gene expression has not been studied in *M. truncatula* SE. In *de novo* shoot regeneration investigations in *Arabidopsis* in response to cytokinin, there was reduced DNA methylation, increased levels of histone H3K4me3 and H3k9ac, and reduced levels of H3Kme2 at the *WUS* sequences (Li et al., 2011).

Expressing slightly later than *WUS* is the *KNOX* gene *SHOOT MERISTEMLESS* (*STM*) (Orłowska and Kępczyńska, 2018) which in zygotic embryogenesis in *M. truncatula* has a similar time course of expression to *WUS* (Kurdyukov et al., 2014b). In *Arabidopsis*, STM is required for continued stem cell function in the SAM by sustaining expression of *WUS* (Scofield et al., 2014).

### Initiating an Embryogenic Program

**Figure 5:**
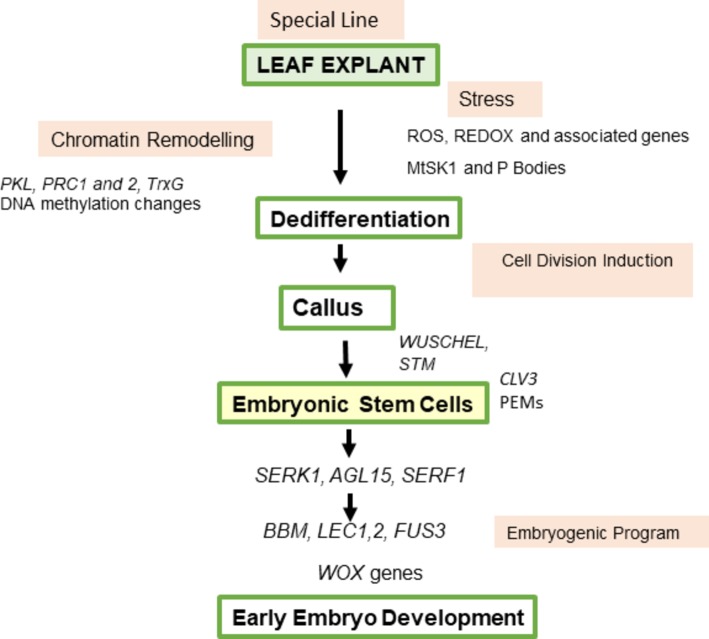
Model for sequence of events in somatic embryogenesis from *M. trunctula* leaf explants. Excision and plating of the explant produce ROS that probably in interaction with hormones cause chromatin remodeling involving *PKL*, *PRC* complexes, and *TRITHORAX* genes, and a stress kinase that maybe linked to transcript degradation *via* RNA processing bodies. Cell divisions follow and callus is produced. Cytokinin-dependent *WUS* expression is essential for SE and there is an initial expression analogous to that occurring in the ovule followed by stem cell development linked to PEMs in patches on the callus, correlating with *CLV3* and *STM* expression. AGL15, SERK, and SERF are part of the correct hormone milieu leading to expression of *BBM* and *LEC* genes and the embryogenic program. The changes diagramed go hand in hand with hormonal changes. Auxin is initially high then lowers as differentiation starts, followed by auxin regulation associated with embryo patterning in the formation of the bipolar embryo. Cytokinin is important for specific genes and the cell cycle. Endogenous ethylene is produced and endogenous ABA/GA ratios specific to *M. truncatula* influence SE. Ethylene, ABA, and GA can be involved in regulating auxin responses. Abbreviations are in the text.

For the embryonic stem cells to develop into embryos, appropriate hormone and transcription interactions are required (Table 2, Figure 5). Essentially upstream of the *LEC* gene transcription, there are the *SERK1*, *AGL15*, *SERF1*, and *BBM* genes.

There are interesting interactions between SERK1, AGL15, and SERF1. MtSERF1 is ethylene dependent, responding to increased ethylene and possibly WUS (Mantiri et al., 2008a). In soybean, AGL15 stimulates *LEC2*, *FUS3*, *ABI3*, and *SERF1* expression (Zheng et al., 2009, 2013). AGL15 also represses the auxin response and interacts with GA metabolism to influence *LEC* genes (Zheng et al., 2016). In relation to the auxin response, a common classic response to auxin and SE is that high auxin is required for the initiation of SE and then auxin removal for embryo development (Halperin, 1964; Rose, 2004). In the model proposed by Fehér (2015), removal of 2,4-D blocks cell proliferation and triggers differentiation. Later, endogenous auxin is produced as part of normal embryogenesis paralleling zygotic embryogenesis. Ethylene and ERF genes are potentially capable of reducing the GA response through DELLA interactions (Achard et al., 2007; Marín-de la Rosa, 2014; Zheng et al., 2016). There is also evidence for a connection between AGL15 and SERK1. AGL15 has been found in the same complex with SERK1, supporting an involvement in the same signaling pathway (Karlova et al., 2006). It is possible that these interactions with SERK1, AGL15, and SERF1, and their relationship to hormone effects, provide the milieu for the activation of SE genes.

The key roles of the leafy cotyledon genes in SE are well established (Lotan et al., 1998; Stone et al., 2001; Gaj et al., 2005; Braybrook and Harada, 2008). As master regulators of embryogenesis (Santos-Mendoza et al., 2008), it is the *LEC1*, *LEC2*, and *FUS3* genes that finally need to be switched on to set in train the embryogenic program. As indicated here, and in other reviews, the leafy cotyledon genes are expressed downstream of WUS (Fehér, 2015). An argument can be made that 35S/LEC1 and 35S/LEC2 seedlings produce somatic embryos (Lotan et al., 1998; Stone et al., 2001) because of the presence of pre-existing stem cells that exist in the vascular and apical meristems. It has been shown that LEC2 can stimulate local auxin synthesis *via YUCCA* genes in *Arabidopsis* (Stone et al., 2008; Wójcikowska et al., 2013) and this is consistent with regulating hormone auxin levels required for embryo development (Friml et al., 2003). Overexpression of *BABY BOOM* (*BBM*), like *LEC* genes, can induce SE (Boutilier et al., 2002) and recent investigations place *BBM* upstream of *LEC* genes and part of the same SE pathway (Horstman et al., 2017b). This is consistent with the Table 2 timeline of *M. truncatula* gene expression. That the cell context is important was shown in the *BBM* studies where overexpression at different stages of germination caused differences in the way embryos were produced, with and without a callus phase.

The overexpression of *WUS* can also induce SE in *Arabidopsis* (Zuo et al., 2002). Again, pre-existing cells and the degree of stemness could be key as to the cells that respond. *WUS* expression is upstream of *BBM* in *M. truncatula* (Chen et al., 2009; Orłowska et al., 2017; Orłowska and Kępczyńska, 2018). Ectopic expression of *AtWUS* produced embryogenic callus in cotton (Zheng et al., 2014), but not regeneration, and resulted in up-regulation of *GhLEC1*, *GhLEC2*, and *GhFUS3. Overexpression of WUS in Coffea canephora* also increased SE (Arroyo-Herrera et al., 2008). In a number of monocotyledons, overexpression of both *WUS* and *BBM* initiates high rates of SE (Lowe et al., 2016). In addition to their established roles in the induction of SE in *Arabidopsis* and *M. truncatula, WUS* (Mayer et al., 1998; Chen et al., 2009) and BBM (Imin et al., 2007; ten Hove et al., 2015) have pivotal roles in the SAM and RAM (root apical meristem) respectively, which suggests that SE is able to co-ordinate these zygotic embryogenesis roles in the SE induction phase. Some years ago, work on *M. sativa* based on intercrossings indicated that two genes were important in SE determination (Reisch and Bingham, 1980; Hernandez-Fernandez and Christie, 1989; Kielly and Bowley, 1992). This suggests that in recalcitrant legume varieties, including the *Medicago* genus, overexpression of key genes is worth further investigation.

Once embryos start to develop, then *WOX* genes become important and the controls characteristic of zygotic embryogenesis follow (Kurdyukov et al., 2014b; *ten Hove et al.,* 2015).

One aspect that also requires further investigation comes from microarray data in the study by Kurdyukov et al. (2014a) where 28 d cultures showed up-regulation of a number of genes that are also linked to nodulation.

In the case of indirect callus-based SE in *M. truncatula,* the following model is suggested, based on current understanding (Figure 5).

## Conclusions

The hormonology and stress responses for SE are characteristic of different species and cultivars, but the principles illustrated in *M. truncatula* provide a basis for understanding indirect callus-based SE from this legume model (Figure 5). Current data (Kurdyukov et al., 2014a) with *M. truncatula* indicate that the special lines required for SE have epigenetic changes but at this stage which genes are critical have not been ascertained. This is an important question for further work, as are epigenetic change and stress (Grafi and Barak, 2015). The *M. truncatula* studies provide some insights into how exogenous hormones (auxin and cytokinin) and endogenous hormones (ABA, GA, and ethylene) contribute to different SE components and their integration. Some of the work with *PRC1*, *PRC2,* and *TRX* genes and *MtSK1* and P-bodies suggest ways to explore chromatin remodeling and dedifferentiation on the way to cell division initiation. Overexpression and gene knockdown studies to assist in more fully defining the sequence of events and networks responsible for SE in *M. truncatula* are required. The very earliest changes involving ROS suggest that what happens at the cell membrane is also an area requiring detailed exploration.

## Author Contributions

The author confirms being the sole contributor of this work and has approved it for publication.

### Conflict of Interest Statement

The author declares that the research was conducted in the absence of any commercial or financial relationships that could be construed as a potential conflict of interest.
